# Associations Between Nutritional Status and Health-Related Quality of Life Among Long-Term Care Residents in Helsinki

**DOI:** 10.1007/s12603-019-1182-1

**Published:** 2019-03-21

**Authors:** Karoliina Sofia Salminen, M.H. Suominen, H. Soini, H. Kautiainen, N. Savikko, R.K.T. Saarela, S. Muurinen, K.H. Pitkala

**Affiliations:** 1 University of Helsinki, Department of General Practice and Primary Health Care, Helsinki, Finland; 2 Vantaa Social Welfare and Health Care, Vantaa, Finland; 3 City of Helsinki, Department of Social Services and Health Care, Developmental and Operational Support, Helsinki, Finland; 4 City of Helsinki, Department of Social Services and Health Care, Oral Health Care, Helsinki, Finland; 5 Helsinki University Hospital, Unit of Primary Health Care, Helsinki, Finland; 6 Helsingin Yliopisto, Helsinki, Finland

**Keywords:** Nutrition, MNA, health-related quality-of-life, nursing home, long-term care

## Abstract

**Objectives:**

We evaluated the associations between nutritional status and health-related quality-of-life (HRQoL) among older long-term care residents in Helsinki.

**Design and participants:**

All 3767 older (≥65 years) long-term care residents in Helsinki in 2017 were invited to participate in this cross-sectional study. After refusals and exclusions of residents without sufficient information, 2160 residents remained.

**Measurements:**

Data on characteristics, nutritional status (Mini Nutritional Assessment, MNA) and HRQoL (15D) were collected by trained nurses.

**Results:**

Of the participants, 64% were at-risk of malnutrition and 18% suffered from malnutrition. Residents in the “malnourished” group were more dependent in activities of daily living (ADL) functioning, suffered more often from dementia, had lower cognitive level, used less medications, and were eating more often inadequately. HRQoL was statistically significantly associated with MNA total score in both female and male residents. There was a curvilinear correlation between MNA and 15D score in females: 0.50 (95% CI 0.46 to 0.53) and males: 0.56 (95% CI 0.50 to 0.61). In partial correlation analysis, all dimensions of 15D, except for sleeping and breathing, were positively associated with MNA score. In these analyses no significant differences emerged between males and females when the results were adjusted for age and dementia.

**Conclusions:**

Nutrition plays an important role in HRQoL among older long-term care residents.

## Introduction

About 14-21% of nursing home residents have suffered from malnutrition when measured with the Mini Nutritional Assessment (MNA) tool (1). The relationship between nutrition status and health-related quality of life (HRQoL) among older people has not been extensively studied. In home-dwelling older people poorer nutritional status has been associated with poorer HRQoL (2, 3, 4, 5, 6). However, studies exploring the relationship between nutritional status and HRQoL are scarce in institutional settings. In an early study, a low body-mass index (BMI) indicative of protein-caloric malnutrition was associated with poor quality of life (QoL) (7). Better nutritional status has been shown to be associated with better psychological wellbeing (PWB) among nursing home residents (8).

HRQoL has been considered a key goal for care of long-term residents (4). HRQoL is designed to measure self-perceived health, encompassing the physical, functional, social and emotional well-being of an individual (4). Depression is known to be associated with decreased HRQoL among older residents of nursing homes (2, 9, 10, 11, 12, 13). High independence in activities of daily life (ADL) has been associated with higher HRQoL in some (14, 15), but not all studies (11). Malnutrition is a risk factor for deterioration of functional status in older adults, which is considered one dimension of HRQoL (4, 7). Thus, nutritional status may be a mediator between functional status and HRQoL.

Thus, there is a paucity of research exploring the relationship between nutritional status and HRQoL according to gender in institutionalized residents. To our knowledge, no studies have investigated which dimensions of HRQoL are associated with poorer nutritional status. The aim of this study was to explore the association of Mini-Nutritional Assessment (MNA) score with HRQoL according to a 15D measure in long-term care residents aged over 65 -years living Helsinki, Finland. In addition, we examined which dimensions of HRQoL are associated with nutritional status in males and females.

## Methods

### Study sample

The study was designed to obtain a comprehensive picture of nutritional status, nutritional care, and related factors of older residents in institutional settings. We collected cross-sectional data in all long-term care facilities (nursing homes and assisted living facilities) in Helsinki, Finland. Assisted living facilities in Finland provide 24-hour care with a registered nurse in charge of the unit. The residents are similar to those in nursing homes, but the environment is more home-like. Altogether 54 long-term facilities with 3767 eligible residents participated in the study. The inclusion criteria were: age ≥ 65 years, living in long-term care, and sufficient information availability on demographic factors, MNA, and HRQoL. Altogether 2545 (68%) residents provided informed consent. Of these, 2160 had data on both MNA and 15D available and were included. The dropouts were either refusals or residents with dementia having no proxy to give informed consent.

### Measurements

Data were collected by registered nurses in March 2017. In each ward, a thoroughly trained nurse assessed residents’ nutritional status by the MNA and retrieved demographic information, diagnoses, and use of medications from medical records. They assessed HRQoL according to the 15D instrument.

The MNA gives a maximum score of 30 points. Less than 17 points indicates malnutrition, 17–23.5 risk of malnutrition, and 24 or more good nutrition status (16, 17). The MNA is validated and widely used internationally (17). In addition, each resident was weighed and body mass index (BMI) calculated as weight divided by height squared (kg/m2).

The 15D instrument is a validated, generic measure for HRQoL (18). The dimensions of 15D are mobility, vision, hearing, breathing, sleeping, eating, speech, excretion, usual activities, mental function, discomfort and symptoms, depression, distress, vitality and sexual activity. 15D can be completed during an interview with the subject or completed by proxy. It combines the advantages of a profile and a preferencebased, single-index measure (18). Score 0 indicates the poorest HRQoL and 1 indicates perfect HRQoL. The 15D scores are reliable, sensitive and responsive to change (18). The nurse most familiar with resident was interviewed if the subject was cognitively impaired. The 15D score has been calculated from all dimensions of the instrument. In this study, all participants responded to the question concerning sexuality “My state of health makes sexual activity almost impossible”. Thus, this question was excluded from the partial correlation analysis.

Comorbidities were evaluated with the Charlson Comorbidity Index (CCI), which takes into account both the number and severity of a person’s medical conditions. High score indicates a greater burden from comorbidities (19).

Cognitive functioning and dependence in ADL were assessed with questions retrieved from the Clinical Dementia Rating Scale (CDR). The “memory” question was used to evaluate the resident’s stage of cognition (0=no memory problems, 0.5=possible memory problems, 1=mild problems, 2=moderate problems, or 3=severe problems) and was categorized into two groups: those with CDR <2 and those with CDR ≥2. The resident’s dependence in ADL functions was evaluated by CDR “personal care” question (1=totally independent; 2=needs prompting, 3=requires assistance in dressing, personal hygiene, and keeping of personal belongings, 4=requires much help with personal care; often incontinent). CDR “personal care” ≥ 2 was defined as dependence in ADLs.

The use of snacks was inquired as follows “Does the resident eat snacks between meals?” (yes/no). The amount of offered food eaten by the resident was evaluated with the question “How much does the residents on average eat from the main meal?” with the five options, “eats only a little, eats less than half, eats half their meal, eats most of their meal or eats all or nearly all of their meal”. The nurses were informed to use images of model portions to estimate this. The responses “eats only a little and eats less than half” were dichotomized as eats inadequately and the response “eats half their meal, eats most of their meal or eats all or nearly all of their meal” as eats adequately.

The local ethics committee of Helsinki University Hospital approved the study. An informed consent was acquired from all participants or in case of moderate-severe dementia from their closest proxies.

### Statistical analysis

The descriptive statistics are presented as means with SDs or as counts with percentages. Statistical significances for the unadjusted hypothesis of linearity across categories of MNA (<23.5, 17.-23.5 and <17) were evaluated by using the Cochran-Armitage test for trend and analysis of variance with an appropriate contrast. In the case of violation of the assumptions (e.g. non-normality), a bootstrap-type test was used. The relationships of MNA and 15D-score was evaluated by use of quadratic model (curvilinear correlation). Adjusted correlation (partial) coefficients MNA and 15D dimensions were calculated by the Pearson method, using Sidak adjusted probabilities. The normality of the variables was tested by using the Shapiro–Wilk W-test. Stata 15.1 (StataCorp LP, College Station, TX, USA) was used for the analysis.

## Results

The mean age of participants was 84 years, and 73% were females. According to MNA, 64% were at-risk of malnutrition and 18% suffered from malnutrition. Residents in the “malnourished” group were more often females, had lower BMI, and suffered more often from dementia. They had lower a level of cognition according to CDR, less medications, and were more dependent in ADL functioning. They ate more often inadequately. Characteristics of the study sample according to nutrition stage are provided in Table 1.

**Table 1:** The Study population’ characteristics according to nutrition status

**Variable**	**Normal nutritional status (< 23.5 points) n=395**	**Risk of malnutrition (17 - 23.5 points) n=1383**	**Malnutrition (< 17 points) n=382**	**P-value**^1^
Female, n (%)	272 (69)	1021(74)	292 (76)	0.016
Age, mean (SD^2^)	83 (8)	84 (8)	85 (8)	0.15
Education (< 8 years), n (%)	151 (41)	528 (42)	142 (44)	0.42
Charlson^3^, mean (SD)	2.0 (1.4)	2.1 (1.3)	2.0 (1.3)	0.71
Dementia, n (%)	262 (66)	1094 (79)	324 (85)	<0.001
Stroke, n (%)	83 (21)	344 (25)	82 (21)	0.86
Number of medications, mean (SD^2^)	9.8 (3.6)	8.9 (3.5)	8.1 (3.6)	<0.001
CDR^4^, memory item >2, n (%)	134 (35)	822 (59)	287 (75)	<0.001
CDR^4^, personal care item >2, n (%)	283 (74)	1245 (92)	368 (99)	<0.001
BMI^5^, mean (SD)	27.9 (4.6)	25.4 (4.7)	20.0 (3.6)	<0.001
Eats snacks between meals, n (%)	307 (78)	1030 (76)	276 (73)	0.13
Eats inadequately, n (%)	37 (9)	297 (22)	171 (45)	<0.001


1. P for linearity was evaluated by using the Cochran-Armitage test for trend and analysis of variance with an appropriate contrast; 2. SD=Standard deviation; 3. Charlson comorbidity index (Charlson et al. 1987); 4. CDR=Clinical Dementia Rating (Hughes et al. 1982); 5. BMI=body mass index (kg/m2)

The mean 15D score was 0.61 (SD 0.13) among females and 0.62 (SD 0.14) among males. Male residents had better HRQoL than female residents (p=0.020). Among females with a normal nutritional status the mean 15D score was 0.71 (SD 0.12), among those at-risk of malnutrition 0.61 (SD 0.12), and among malnourished residents 0.52 (SD 0.10). Among males with a normal nutritional status, the mean 15D score was 0.74 (SD 0.11), among those at-risk of malnutrition 0.61 (SD 0.12), and among malnourished residents 0.51 (SD 0.10).

**Figure 1:**
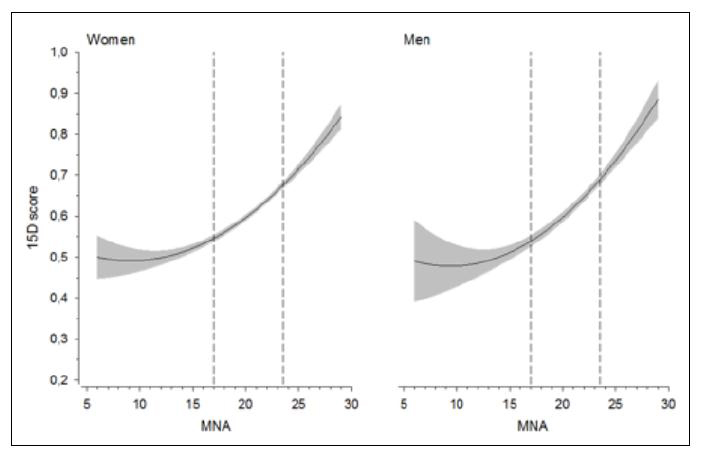
Relationship between Mini Nutritional Assessment (MNA) Score (16) and health-related quality-of-life according to 15D (18) score in females and males. Curvilinear correlation with 95% confidence intervals (in gray). The dashed lines represent the cut-off points for malnutrition (<17 points in MNA), at-risk of malnutrition (17 - 23.5 points) and normal nutritional status (>23.5 points)

There was a curvilinear correlation between MNA and 15D score (Figure 1). The curvilinear correlation among men was 0.56 (95% Cl 0.50 to 0.61) and among women 0.50 (95% 0.46 to 0.53) (Figure 1). The 15D score started to increase among those at-risk of malnutrition, and it continued to increase linearly among those who were well-nourished. HRQoL was significantly associated with MNA score in both female and male residents.

**Figure 2:**
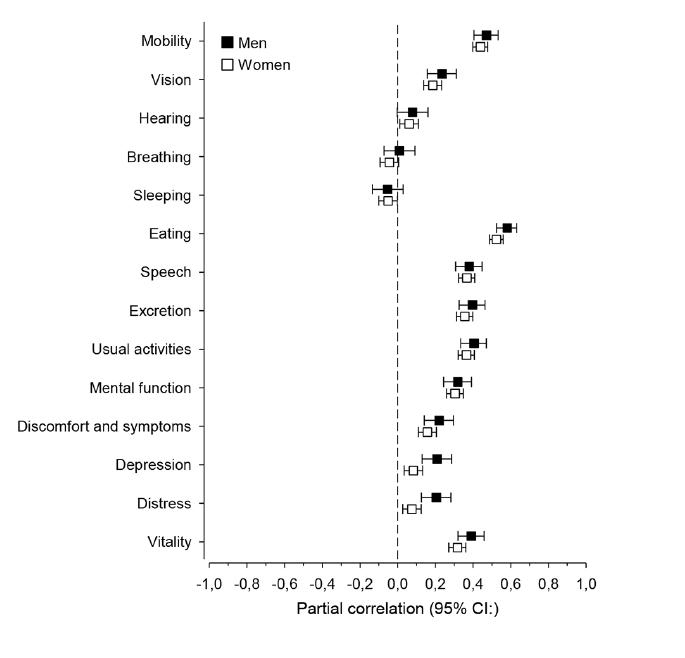
Partial correlation with 95% confidence intervals between dimensions of 15D (18) and Mini Nutritional Assessment (MNA) total score (16) in male and female residents. Adjusted for age and dementia

Results from the partial correlation analysis between the MNA total score and the 14 dimensions of the 15D according to gender are presented in Figure 2. All dimensions of the 15D, except for sleeping and breathing, were associated with the MNA score in both genders. The strongest correlations for the 15D dimensions were found in “eating” and “mobility” in both genders. No significant differences emerged between males and females in partial correlations of the 14 dimensions of 15D with MNA total score after adjustment for age and dementia.

## Discussion

Nutritional status was significantly associated with HRQoL among institutionalized residents of both sexes. A curvilinear correlation existed between MNA and 15D score in such way that 15D score had a “floor effect” among those who were severely malnourished. The 15D score increased rapidly in a linear manner among those who were at-risk of malnutrition or had normal nutritional status. Of dimensions of 15D, mobility and eating had the strongest partial correlation with MNA, whereas sleeping and breathing did not have a significant association with MNA. There were no significant differences between males and females in partial correlation of the 14 dimensions of the 15D with MNA total score after adjustment for age and dementia.

Strength of this study are large sample size and the fairly good representativeness of the sample. All residents who were residing in nursing homes and assisted living facilities in the city of Helsinki were invited to participate. However, the response rate was only 68%. Of these, 85% had both MNA and 15D scores available. A limitation of the study is its cross-sectional nature. Thus, whether HRQoL has a causal relationship with nutritional status cannot be concluded. However, to our knowledge, there are no previous studies exploring the relationship between MNA and HRQoL in nursing homes. Furthermore, the associations between different dimensions of HRQoL and nutrition according to gender have not been explored in earlier.

Previous research has revealed that malnutrition is common among aged residents in nursing homes (1, 20, 21, 22, 23). In the metaanalysis the prevalence of malnutrition varied between 14% and 21% in nursing homes (1). In our study, the prevalence of malnutrition was 18%. Educational attempts have been made since 2003 to improve nutritional care in nursing homes in Helsinki (24, 25). Finnish nutritional guidelines were published in 2010 (26). An improvement has taken place despite increasing disabilities among institutionalized residents (25). Residents’ mean age is higher and they are more disabled now than ten years ago (25). Many comorbidities, high age and increased disability should increase the risk of malnutrition (1). Lower BMI, decreased level of cognition, lower number of medication and dependency in ADL were associated with malnutrition. These observations are in line with previous studies (20).

Our results are consistent with earlier studies exploring the association of nutrition status with HRQoL or psychological well-being. In both community-living and non-institutionalized older people, malnutrition has been associated with poor HRQoL (27, 28, 29).

In previous studies HRQoL has been poorer in females than in males among non-institutionalized and communityliving individuals (3, 27, 30). One explanation for the gender differences in HRQoL could be that females more willingly report health problems than males and they have higher expectations of health and function (27).

Impairments in ADL – an important dimension of HRQoL - are associated with reduced QoL in nursing home residents (31, 32). The impairments in ADL has been associated with decline in QoL in several studies (10, 14, 33, 34). In our study, impairments in ADL in the 15D dimensions of mobility, excretion, usual activities, and even eating were associated with the MNA score. As items in both MNA and 15D measure ADL dimensions, it is not unexpected that nutrition stage and HRQoL were associated. However, these findings do not explain why the curvilinear correlation between the MNA and 15D was so strong.

In addition to ADL impairments, mental function in 15D indicating cognition was also significantly correlated with the MNA score. This is also understandable since cognitive stage was associated with nutritional status. Both dementia and depression are included in one item of the MNA. It is obvious that eating and vitality were associated with the MNA score.

No association existed between breathing or sleeping and the MNA score. There may be other confounders affecting these dimensions besides the MNA. However, all of the factors that predict HRQoL in long-term care residents are not wellknown. The causality between nutrition status and HRQoL needs further studies. Nutrition intake and nutritional status are potentially mediators between functional status and HRQoL.

## Conclusions

Nutritional status according to the MNA is significantly associated with HRQoL in both male and female residents in institutional settings. Our study emphasizes that nutrition is an very important element in maintaining HRQoL. Additional means to support nutrition in older people should be studied.
